# Content of industrially produced trans fatty acids in breast milk: An observational study

**DOI:** 10.1002/fsn3.2862

**Published:** 2022-04-05

**Authors:** Christian Mayela Bousset‐Alféres, Jorge Luis Chávez‐Servín, Pedro Alberto Vázquez‐Landaverde, Claudia Azucena Betancourt‐López, María del Carmen Caamaño, Roberto Augusto Ferriz‐Martínez, Elsa Fernanda Chávez‐Alabat, Ma Guadalupe Lovatón‐Cabrera, Karina de la Torre‐Carbot

**Affiliations:** ^1^ Facultad de Ciencias Naturales Universidad Autónoma de Querétaro Querétaro México; ^2^ Centro de Investigación en Ciencia Aplicada y Tecnología Avanzada (CICATA) Instituto Politécnico Nacional Querétaro México; ^3^ Banco de Leche, Secretaría de Salud del Estado de Querétaro (SESEQ) Querétaro México

**Keywords:** breast milk, elaidic acid, essential fatty acids, industrially produced trans fatty acids

## Abstract

Breast milk may contain industrially produced trans fatty acids (TFAs), which can affect the content of essential fatty acids (EFAs). This could have significant implications for the child's development. The fatty acids present in breast milk can be modified by adjusting the mother's diet. The objective of this study was to determine the content of industrially produced TFAs present in colostrum, transitional milk, and mature milk produced by mothers between 18 and 45 years of age in the state of Querétaro, Mexico, based on a longitudinal observational study. The TFA content in the breast milk of 33 lactating women was analyzed using gas chromatography. The mothers’ consumption of TFAs was also estimated by analyzing a log prepared through 24‐hr dietary recall (24HR) obtained in each period. The TFA content in the mothers’ diet was similar across the colostrum, transitional milk, and mature milk phases: 1.64 ± 1.25 g, 1.39 ± 1.01, and 1.66 ± 1.13 g, respectively. The total TFA content was 1.529% ± 1.648% for colostrum; 0.748% ± 1.033% for transitional milk and 0.945% ± 1.368% for mature milk. Elaidic acid was the TFA in the highest concentration in all three types of milk. No correlation was found between the content of industrially produced TFAs in breast milk and the anthropometric measurements of the mother or between the estimated consumption of TFAs and the content of TFAs in breast milk. Elaidic acid and total content of TFAs were negatively correlated (*p* < .05) with the content of docosahexaenoic acid (DHA) (0.394 ± 0.247) (*R* = −0.382) in colostrum. The concentration of TFAs was found to correlate with the composition of EFAs in milk.

## Graphical abstract

Breast milk may contain industrially produced trans fatty acids (TFAs). The objective of this study was to determine the content of industrially produced TFAs in colostrum, transitional, and mature milk. Elaidic acid was the TFA with the highest concentration in all three types of milk. TFA concentrations were significantly higher in colostrum than in transitional or mature milk. Elaidic acid and total TFA content showed an inverse correlation with DHA in colostrum. The concentration of TFAs correlates with the presence of EFAs in human milk.

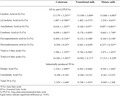

## 1 INTRODUCTION

Breast milk is the ideal food for the newborn (Andreas et al., 2015). Its nutrients and components are essential for promoting proper growth and development of the infant (Andreas et al., 2015; German & Dillard, 2006; Koletzko et al., 2011). The composition of breast milk is variable and may change according to dietary, genetic, sociodemographic, health, and environmental factors (Burianova et al., 2019; Miliku et al., 2019). It also varies over the course of the postpartum period, when different types of milk are produced in succession. These can be classified into three stages of production: colostrum, transitional milk, and mature milk.

The fat present in breast milk is the infant's main source of energy (50% of total energy) (Marín et al., 2009; Vásquez‐Garibay, 2016; Wan et al., 2010). This fat consists primarily of saturated (42%–47%) and unsaturated (53%–58%) fatty acids (Macías et al., 2006; Marín et al., 2009). While 60%–70% of the fat in milk is the result of tissue synthesis and maternal fat deposits, approximately 30% of the fat in breast milk is derived from her diet (De Souza Santos da Costa et al., 2016; Deng et al., 2018). The concentration of fatty acid in breast milk depends on the mother's intake of lipids and is therefore linked to the type of diet prevalent in the region where she lives (Deng et al., 2018; Gómez‐Cortés & De La Fuente, 2017; Marín et al., 2009; Mazzocchi et al., 2018; Perrin et al., 2018; Samur et al., 2009; Wan et al., 2010). The essential fatty acids (EFAs) contained in breast milk are precursors of long‐chain polyunsaturated fatty acids (LC‐PUFAs) and play a key role in the growth and maturation of the central nervous system and development of the infant's visual‐sensory system. Alterations in its concentration may cause damage to the newborn's cognitive and visual development, causing dysfunction and permanent negative effects on IQ and visual acuity (Birch et al., 2005; 2010; De Souza Santos da Costa et al., 2016; Deng et al., 2018; Gómez‐Cortés & De La Fuente, 2017; Jensen et al., 2010; Nishimura et al., 2014; Spector & Kim, 2015; Vega et al., 2012). Human milk may contain trans fatty acids (TFAs), which are unsaturated fatty acids with at least one double bond in the trans configuration (Fernández‐Michel et al., 2008; Islam et al., 2019; Wu et al., 2017). A TFA concentration of 2%–5% has been reported in human milk (Daud et al., 2013; Krešić et al., 2013; Larqué et al., 2001; Samur et al., 2009; Valenzuela, 2008). The human body does not synthesize trans fats, so their presence in breast milk is related solely to maternal intake (Samur et al., 2009). These TFAs may be either naturally or industrially produced. It is estimated that only 5% of total trans fats in the human diet are natural in origin (Ballesteros‐Vásquez et al., 2012). Recent studies have found benefits for the organism from consuming natural TFAs (Kuhnt et al., 2015), while consumption of industrially produced TFAs has been found to have negative health implications (Ginter & Simko, 2016; Islam et al., 2019; Sauvat et al., 2018). Industrially produced TFAs are created through the hydrogenation of vegetable oils in industrial food processing. This is done to improve stability and lengthen shelf life, reduce the cost of these products, and produce desirable organoleptic characteristics for the consumer (Ballesteros‐Vásquez et al., 2012; Castro‐Martínez et al., 2010; Hyseni et al., 2017). The predominant industrially produced TFA in the food supply is elaidic acid (De Souza et al., 2015; Hauff & Vetter, 2009; Krešić et al., 2013; Villalpando, 2007), which has been found in greater proportion in breast milk (Duran & Masson, 2010). However, other industrially produced TFAs have also been identified in breast milk, such as linoelaidic acid (C18:2n6t) (De Souza Santos da Costa et al., 2016; Deng et al., 2018; Moltó‐Puigmartí et al., 2011), petroselaidic acid (C18:1n6t) (De Souza Santos da Costa et al., 2016; Gómez‐Cortés & De La Fuente, 2017), and palmitoelaidic acid (C16:1n9t) (De Souza Santos da Costa et al., 2016; Deng et al., 2018; Gómez‐Cortés & De La Fuente, 2017); as well as isomers C18:1 *t*10 (De Souza Santos da Costa et al., 2016; Duran & Masson, 2010; Gómez‐Cortés & De La Fuente, 2017), C18:1 *t*8, C18:1 *t*11 (De Souza Santos da Costa et al., 2016), C18:1 *t*12 (Gómez‐Cortés & De La Fuente, 2017), C18:1 *t*13, C18:1 *t*14 (De Souza Santos da Costa et al., 2016; Gómez‐Cortés & De La Fuente, 2017), and C14:1 *t* (Perrin et al., 2018). There is evidence that industrially produced TFAs interfere with the organism's ability to metabolize EFAs, because they compete for the same enzymes that desaturate them (∆5 and ∆6 desaturases), blocking the activity of these enzymes in synthesizing LC‐PUFAs, which may have significant implications in newborns (Krešić et al., 2013; Kummerow, 2009; Villalpando, 2007). In fact, the presence of TFAs in milk could trigger an inadequate supply of EFA in human milk (De Souza Santos da Costa et al., 2016; Krešić et al., 2013).

Given their negative health effects, Institutions such as the World Health Organization (WHO) and the Mexican Ministry of Health have recommended that consumption of trans fats be limited to 1% of the total caloric value of a person's diet at most (De Souza Santos da Costa et al., 2016; Downs et al., 2017; Kuhnt et al., 2015; Secretaría de Salud, 2013). However, according to a report by the Global Burden of Diseases Nutrition & Chronic Diseases Expert Group, 2014, the trans fats consumed by Mexicans account for 3.6% of their caloric intake on average, making this one of the highest trans fat‐consuming countries in the world (Global Burden of Diseases Nutrition & Chronic Diseases Expert Group, 2014). The average global intake is 1.4% (Downs et al., 2017; Global Burden of Diseases Nutrition & Chronic Diseases Expert Group, 2014).

The objective of the present study was to determine the content of industrially produced TFAs in colostrum, transitional milk, and mature milk produced by mothers 18–45 years of age, from the state of Querétaro, Mexico.

## 2 MATERIALS AND METHODS

### 2.1 Study type and spatiotemporal location

A longitudinal observational study was carried out. Study participants were recruited at the Hospital for Specialties of Children and Women between November 2017 and March 2018. Samples were processed at the Faculty of Natural Sciences of the Autonomous University of Querétaro (UAQ). The fatty acid determinations were made at the Center for Research in Applied Science and Advanced Technology of the National Polytechnic Institute (CICATA).

### 2.2 Participants

The sample consisted of lactating women who had given birth at the Hospital for Specialties of Children and Women. Participants were recruited in the short‐term Postnatal and Gynecology areas. The inclusion criteria were: clinically healthy women who had given birth to a single child at most 5 days prior to recruitment; who were breastfeeding; aged between 18 and 45 years; and who agreed to participate in the study. The sample excluded mothers who had undergone some kind of drug therapy in the previous 7 days or hormonal therapy in the previous three weeks; those who reported consuming some psychoactive drug during pregnancy or lactation; those who reported some impediment to breastfeeding; those who consumed any nutritional or dietary supplement that contained fatty acids during the lactation period, or those who declined to continue in the study. Initially, 56 women were included in the study, 33 of whom completed the three milk samples.

### 2.3 Study design and procedures

Each mother was visited three times. During these sessions, anthropometric measurements were taken, and samples of breast milk collected.

The first session was carried out during the colostrum production period (days 1–5 after delivery); the second during the production of transitional milk (days 6–15 after delivery) and the third during the production of mature milk (from day 15 to the end of the first month postpartum).

In the first session, after informed consent was obtained, the birth weight of the newborn was taken from the mother's file and the following questionnaires were applied to the mother: medical history, which included general information and personal pathological, hereditary–family, and gynecological/obstetrical histories; socioeconomic study questionnaire; food surveys; and the infant questionnaire, through which the data on the lactation pattern and the health status of the infant were recorded. In the remaining two sessions, the infant questionnaire and feeding surveys were also applied.

#### 2.3.1 Collection of breast milk samples

Breast milk was expressed manually by the mother based on the Official Mexican Standard NOM‐043‐SSA2‐2012, Basic Health Services: Promotion and education for health in alimentary matters, published by the Mexican Ministry of Health (Secretaría de Salud, 2013), which provides criteria for guidance and assistance if needed. Colostrum extraction was carried out at the Hospital for Specialties of Children and Women, and the transitional milk and mature milk were extracted at the nursing mother's home.

Between 2 and 5 ml of colostrum was collected in each extraction, and from 10 to 30 ml of transitional milk and mature milk. The extraction was carried out between 8:00 a.m. and 5:00 p.m. and was obtained in all cases from a single breast.

Once the sample was collected, the milk was labeled with the mother's name, date of extraction, weeks of gestation of the baby, time of birth of the baby, and volume of milk provided. It was then placed in a cooler with prefrozen gel and transported to the Cell Biology and Human Nutrition laboratories of the Faculty of Natural Sciences, UAQ, Juriquilla campus, where it was stored in a freezer at −80°C for subsequent analysis.

#### 2.3.2 Food surveys and analysis of the maternal diet

The mother's diet was evaluated by analyzing the 24‐hr dietary recall (24HR), a structured interview used to capture detailed information on all the foods and beverages consumed in the past 24 hr. During each visit to the mother, three such records were collected: two pertaining to weekdays and one to a weekend day. This means that nine 24‐hr logs were collected for each mother: three for the colostrum period, three for the transitional milk period, and three for the mature milk period. The “Food Base of Mexico: Compilation of the composition of foods frequently consumed in the country” (Ramírez‐Silva et al., 2019) and the “NutriNet‐System: calculations for net grams and nutrients in the dietary system” (Ramírez‐Silva & Barragán‐Vázquez, 2020) were used for the calculation of macronutrients and dietary energy consumption. The amount of industrially produced TFAs in the mother's diet was estimated using the fatty acid composition tables of foods consumed frequently in the Mexican diet (Villalpando, 2007). The TFAs present in the mother's diet were quantified and averaged, thus obtaining an estimate of their habitual consumption at each stage of lactation. TFA consumption was expressed in grams and as a percentage of the total caloric value of the diet. The number of kilocalories supplied by TFA per gram was determined, as well as the amount of energy from all the macronutrients consumed, and consumption of TFA was thus calculated as a percentage of the total.

#### 2.3.3 Obtaining anthropometric measurements from the mothers

During the first visit, the mother's height, prepregnancy weight, and maximum weight during pregnancy were obtained from the clinical record. Subsequently, measurements of weight and body fat percentage were taken from the mothers during their hospital stay (first 5 days postpartum: colostrum stage) and at home (day 5 to 15 postpartum: transitional stage; and >15 days postpartum: mature milk stage). An Omron scale and body composition monitor was used, which functions using bioelectrical impedance analysis (OMRON HEALTHCARE INC, 2017). For measurement, the scale was placed on a flat surface and mothers were asked to take off their shoes and remove any heavy clothing before being weighed. The weight was taken in an upright position, with the feet on the electrodes and the arms hanging parallel to the body. The fat percentage was measured in an upright position, with the feet and hands on the electrodes.

### 2.4 Analysis of milk

#### 2.4.1 Reagents and standards

Sodium methylate in methanol (CH3ONa, Merk) was used for the saponification of milk fat. Boron trifluoride solution in methanol (14% w/v) (BF3/MeOH, Sigma‐Aldrich) was used for the esterification of fatty acids, and n‐hexane (Karal) and sodium chloride (NaCl, Fermont) for the extraction of fatty acids. Anhydrous sodium sulfate (Na2SO4, J.T. Baker) was used as a desiccant. Hydrogen (INFRA) was used as a carrier gas for the gas chromatograph. As an internal standard, C13: 0 methyl ester (tridecanoic acid, Sigma) was used; while for the determination of the retention time of the fatty acids, the pure standards FAME Mix C4‐C24 (purity 98.7%–99.9%, Supelco), trans‐6‐Octadecenoic acid methyl ester (trans‐6‐Petroselaidic acid methyl ester, purity 99.0%, Supelco), linoleic Acid Methyl Ester Mix (cis/trans) (purity 98.0%, Supelco), and trans‐9‐Octadecenoic acid (elaidic acid, purity >99%, Supelco) were used.

#### 2.4.2 Equipment

The percentage of body fat of the mothers was measured, using a scale equipped with a bioelectrical impedance monitor (OMRON HEALTHCARE Model HBF‐514C., U.S.A). A multiblock heater (Lab‐Line Multi‐Blok Heater. Model: 2052AB) was used during the extraction of fatty acids from the samples. Fatty acid determination was performed on an Agilent 6890A gas chromatograph (Agilent Technologies) equipped with a fused silica capillary column (SP2560; 100 m × 25 mm diameter with 0.2‐micron film thickness; Supelco, Inc.) and a flame ionization detector (FID).

#### 2.4.3 Fatty acid determinations

The extraction of samples from breast milk for the subsequent determination of TFAs and EFA was carried out using the methodology described by Chávez‐Servín, 2009 (Chávez‐Servín et al., 2009), in the laboratories of the Faculty of Natural Sciences, UAQ. Briefly, the breast milk samples were homogenized, starting with 200 µl of breast milk in each tube; 40 µl of the C13: 0 internal standard solution previously diluted in 1 mg/ml n‐hexane was added to each tube; 1 ml of a sodium methylate solution (CH3ONa) in methanol was added; and after shaking, the test tubes were placed in a multiblock heater preheated to 115°C for 17 min; then subsequently cooled in a water bath. Once at room temperature, 1 ml of boron trifluoride in methanol (BF3/MeOH) (14% w/v) was added to each tube; after stirring, the tubes were placed again in the multiblock heater preheated to 115°C for another 17 min; then they were cooled again to room temperature. Subsequently, they were mixed with 2 ml of hexane and 2 ml of saturated NaCl solution. Subsequently, after centrifugation, 1.5 ml of the hexane phase was transferred to Eppendorf tubes in which a spatula tip with anhydrous sodium sulfate was previously added. After stirring and subsequent resting, the hexane phase was transferred to a vial and stored in the freezer at −20°C until the time of injection into the gas chromatograph.

After the samples were processed, the fatty acids were determined at CICATA using an Agilent 6890A gas chromatograph (Agilent Technologies) equipped with a fused silica capillary column SP2560; 100 m × 0.25 mm diameter with 0.2‐µm film thickness (Supelco, Inc., Bellefonte, PA, USA) and a FID.

The oven program was as follows: temperature of 70°C for 4 min, 8°C/min ramp to 110°C, 5°C/min ramp to 170°C for 10 min, and 4°C/min ramp at 215°C for 23 min. Inlet and detector temperatures were 250°C. The flow rate of the hydrogen carrier gas was 1 ml/min. Peaks based on purified standards were identified: FAME Mix C4‐C24 (purity 98.7%–99.9%, Supelco), trans‐6‐Octadecenoic acid methyl ester (trans‐6‐Petroselaidic acid methyl ester, purity 99.0%, Supelco), linoleic Acid Methyl Ester Mix (cis/trans) (purity 98.0%, Supelco), and trans‐9‐Octadecenoic acid (elaidic acid, purity >99%, Supelco).

### 2.5 Statistical analysis

A descriptive analysis of the population characteristics was carried out to determine the mean, standard deviation, and minimum and maximum values. A comparative analysis of the means of the anthropometric indicators for each mother was carried out at each of the three milk production stages: maternal weight (kg), body fat (%), body mass index (BMI; kg/m^2^), weight lost (%) after delivery, and percentage of prepregnancy weight (%). Likewise, the means of the food variables were compared: energy consumption (Kcal), lipids (%), proteins (%), carbohydrates (%), and industrially produced TFAs in the diet (g, %), by a general linear model. A comparative analysis of the composition of fatty acids in colostrum, transitional milk, and mature milk was carried out, using the Friedman test to compare the corresponding means. The population was classified according to the BMI and percentage of body fat. To compare the content of industrially produced TFAs and EFAs in breast milk according to these two classifications, the Kruskal–Wallis test was used. Since most of the TFA and EFA variables did not conform to a normal distribution, in order to measure the association between “EFA and industrially produced TFAs in breast milk” and “industrially produced TFAs in maternal diet,” several logistic regressions were performed to evaluate the probability of finding TFA or EFA content above the median value in breast milk, when the mother consumes more calories or a higher percentage of each macronutrient. Finally, the association between the “industrially produced TFAs” and “EFA” variables in breast milk was analyzed for colostrum, transitional milk, and mature milk, analyzing R, R^2^. The confidence interval (CI) for all analyses was 95%, with a statistical significance of *p* < .05.

## 3 RESULTS AND DISCUSSION

### 3.1 Sample characteristics

The study involved 56 breastfeeding women. In the end, 33 of these mothers completed the three milk samples and the requested questionnaires, and these constituted the sample used. The mean age of the 33 women was 25.3 ± 6.7 years. They were recruited on their first postpartum day, with an average of 38.8 ± 1.1 weeks of gestation, with an average of 2 children (range 1–5). In relation to marital status, 66.6% of participants were living in a domestic partnership, 18.1% were married, and 15.1% were single. Regarding occupation, participants were employed primarily in home and family care (75.75%), working for others (9.09%), students (6.06%), self‐employed merchants (6.06%), or professionals (3.03%). The birth route of the most recent child was vaginal delivery in 81.81% of participants, and caesarean section in 18.18%. With regard to support for breastfeeding in the hospital, in the first feeding immediately after birth, 84.84% of the infants were breastfed, 3.03% (one infant) initially received formula (and subsequently received breast milk), and 12.12% received mixed feeding (alternating breast milk and formula). As many as 54.54% of mothers fed their babies breast milk within the first hour of birth; most of the mothers were able to be with their baby during their hospital stay (96.96%); while only one mother (3.03%) was separated from her baby due to health conditions. Finally, the socioeconomic level of mothers was middle‐ to very low‐income for more than 90% of the women studied. The largest percentage of participants (60.60%) was in the middle–lower income bracket.

### 3.2 Anthropometric measurements of mother and infant

The infants involved in this study were generally of normal weight (2500–4500 g) at birth, with a mean of 3025.1 ± 462.6 g. However, 12.1% of infants had low birth weight (<2500 g), according to WHO criteria. None of the newborns weighed more than 4500 g at birth.

Maternal height (cm) was 156.4 ± 5.5. The average weight and pregestational BMI were 58.7 ± 12.8 kg and 23.9 ± 4.8 kg/m^2^, respectively. Maximum weight and weight gain in pregnancy were 70.5 ± 11.1 and 11.7 ± 5.1 kg, respectively. According to the pregestational BMI, weight gain in pregnancy was appropriate in 42.42% of women, less than the minimum recommended in 27.27% of the cases, and more than the recommended maximum in 30.30%, according to pregnancy weight gain tables supplied by the Institute of Medicine (Institute of Medicine (US) and Pregnancy, 2009).

Weight, BMI, and percentage of prepregnancy weight were all found in higher ranges during the colostrum stage, which is due to weight gain during pregnancy. However, these values decreased progressively and significantly over the course of the colostrum, transitional milk, and mature milk stages (*p* < .05). These results may be due to the mother's postpartum recovery and the added energy expenditure that breastfeeding implies, which contributes to the return to prepregnancy weight. A significant increase in the percentage of body fat was observed at 5 to 15 days postpartum (transitional stage) compared to the percentage in the colostrum stage. In the colostrum stage, the percentage of mothers with a high percentage of body fat (body fat > 33%) (Gallagher et al., 2000) was lower (39.39%) than in the transitional (48.48%) and mature (54.54%) milk stages. This is probably because the body requires more fat for milk production at each stage (Table 1).

**TABLE 1:** Anthropometric measurements of mothers in the three stages of lactation composition (*n* = 33)

	Colostrum stage	Transitional stage	Mature milk stage
Maternal weight (kg)	66.29 ± 10.42^a,b^	63.06 ± 9.76^a^	62.24 ± 10.55^b^
Body fat (%)	29.98 ± 7.53^a^	33.03 ± 6.17^a^	31.49 ± 7.35
BMI (kg/m^2^)	27.08 ± 4.12ª^,b^	25.76 ± 3.82^a^	25.41 ± 4.07^b^
Weight lost (%)	6.03 ± 2.06ª^,b^	10.53 ± 3.25^a^	11.82 ± 4.63^b^
Pregestational weight percentage (%)	114.23 ± 10.23ª^,b^	108.68 ± 9.40^a^	107.03 ± 9.17^b^

Note

Equal letters indicate significant difference (*p* < .05).

Abbreviation: BMI, body mass index.

### 3.3 Estimation of TFA consumption by nursing mothers

The diet of the 33 women during the three stages of lactation was evaluated. The amount of industrially produced TFAs was estimated, as well as the energy consumption and proportion of fats, proteins, and carbohydrates in their diets (Table 2).

**TABLE 2:** Consumption of energy, macronutrients, and industrially produced TFA by women in each stage of lactation (*n* = 33)

	Colostrum stage (day 1–5) X ± SD	Transitional stage (day 5–15) X ± SD	Mature milk stage (1 month) X ± SD
Energy consumption (Kcal)	2205.20 ± 499.23	2036.31 ± 566.06	2240.31 ± 578.09
Lipids (%)	29.83 ± 6.34	31.37 ± 7.27	32.51 ± 4.74
Industrially produced TFA (g)	1.64 ± 1.25	1.39 ± 1.01	1.66 ± 1.13
Industrially produced TFA in relation to total energy consumption (%)	0.63 ± 0.44	0.63 ± 0.49	0.69 ± 0.48
Proteins (%)	13.07 ± 2.72^a^	15.34 ± 3.27^a^	13.75 ± 2.94
Carbohydrates (%)	57.08 ± 7.84	53.27 ± 8.39	53.73 ± 5.71

Note

Equal letters indicate significant difference (*p* < .05).

Abbreviation: TFA, trans fatty acid.

The average daily consumption of TFAs was 1.56 ± 0.75 g/day, and it accounted for 0.65% ± 0.30% of the total caloric value of the diet. No differences were found in energy intake or in the consumption of lipids, carbohydrates, or TFAs in the three stages. This may be due to the temporal proximity between one evaluation and another (evaluations were made at 1–5 days, at 6–15 days, and at 16–30 days). However, protein consumption was significantly higher (*p* < .05) in the transitional stage (15.34% ± 3.27%) than in the colostrum stage (13.07% ± 2.72%), because in the transitional stage, lactating women increased their consumption of animal proteins such as eggs, chicken, and beef.

The intake of TFAs found in this study contrasts with that published in the report on world consumption of fats, carried out from 1990 to 2010, which indicates that the average consumption of trans fat in Mexico accounts for 3.6% of the total caloric value of the diet (Global Burden of Diseases Nutrition & Chronic Diseases Expert Group, 2014). In this study, consumption was found to be below the maximum intake recommended by the WHO and by the Ministry of Health, which is 1% of the total caloric value of the diet (De Souza Santos da Costa et al., 2016; Downs et al., 2017; Kuhnt et al., 2015; Secretaría de Salud, 2011). These differences may be attributed to recent regulations restricting content of trans fats in industrialized products.

The intake of TFAs in the Mexican study population was 1.56 g/day (0.66%), lower than that reported by women in Turkey (2.16 g/day) (Samur et al., 2009), Croatia (2 g/day) (Krešić et al., 2013), and Brazil (3.07 g/day) (1.23%) (De Souza Santos da Costa et al., 2016); but higher than that reported by lactating mothers in Malaysia (1.27 g/day) (Daud et al., 2013), China (0.16%–0.34%) (Deng et al., 2018), and Canada (0.8 g/day) (Ratnayake et al., 2014).

### 3.4 Fatty acid content in breast milk

Fatty acid content in breast milk was analyzed in three postpartum lactation stages (colostrum, transitional milk, and mature milk), including EFAs and their derivatives, the LC‐PUFAs. The fatty acids analyzed and their retention times according to the purified standard are presented in Table 3.

**TABLE 3:** Fatty acids analyzed in breast milk (*n* = 33) (%)

Fatty acid	RT	Colostrum	Transitional milk	Mature milk
Capric acid (C10: 0)	22.84	0.212 ± 0.362^a,b^	1.276 ± 0.399^a^	1.418 ± 0.685^b^
Undecanoic acid (C11: 0)	24.95	0.034 ± 0.181^a,b^	0.010 ± 0.006^a^	0.024 ± 0.057^b^
Lauric acid (C12: 0)	27.03	1.330 ± 0.672^a,b^	5.751 ± 2.313^a^	5.801 ± 2.647^b^
Tridecanoic acid (C13: 0)	29.04	2.098 ± 2.778^a,b^	0.552 ± 0.313^a^	1.976 ± 4.226^b^
Myristic acid (C14: 0)	30.96	3.427 ± 1.075^a,b^	6.130 ± 2.608^a^	5.763 ± 2.616^b^
Myristoleic acid (C14: 1)	32.33	0.119 ± 0.197^a,b^	0.173 ± 0.066^a^	0.181 ± 0.071^b^
Pentadecanoic acid (C15: 0)	32.80	0.266 ± 0.238	0.230 ± 0.062	0.315 ± 0.262
cis‐10‐Pentadecenoic acid (C15: 1)	34.12	0.070 ± 0.238	0.008 ± 0.021	0.007 ± 0.013
Palmitic acid (C16: 0)	34.56	21.800 ± 3.881^a,b^	20.251 ± 2.385^a^	20.112 ± 7.150^b^
Palmitoleic acid (C16: 1)	35.61	1.604 ± 0.406^a,b^	2.454 ± 0.658^a^	2.255 ± 0.788^b^
Heptadecanoic acid (C17: 0)	36.23	0.402 ± 0.318^a^	0.298 ± 0.062	0.315 ± 0.163^a^
cis‐10‐Heptadecenoic acid (C17: 1)	37.25	0.407 ± 0.668	0.296 ± 0.092	0.383 ± 0.489
Stearic acid (C18: 0)	37.84	7.045 ± 1.972^a,b^	5.130 ± 0.735^a^	5.741 ± 1.526^b^
Elaidic acid (C18: 1n9t)	38.41	1.334 ± 1.699^a,b^	0.503 ± 0.846^a^	0.585 ± 1.042^b^
Oleic acid (C18: 1n9c)	38.70	32.776 ± 6.275	34.373 ± 4.167	31.919 ± 7.845
Linolelaidic acid (C18: 2n6t)	39.34	0.196 ± 0.182	0.246 ± 0.232	0.361 ± 0.535
Linoleic acid (C18: 2n6c)	40.01	13.179 ± 3.247^a,b^	15.540 ± 3.609^a^	15.660 ± 4.085^b^
Arachidic acid (C20: 0)	40.91	0.636 ± 0.887^a,b^	0.356 ± 0.158^a^	0.396 ± 0.330^b^
γ‐linolenic acid (C18: 3n6)	41.49	1.087 ± 0.580^a,b^	1.402 ± 0.573^a^	1.554 ± 0.651^b^
cis‐10‐Eicosenoic acid (C20: 1)	41.65	1.831 ± 0.859^a,b^	0.943 ± 0.358^a^	1.055 ± 0.985^b^
Linolenic acid (C18: 3n3)	42.27	0.699 ± 1.085^a,b^	0.170 ± 0.095^a^	0.665 ± 1.749^b^
Heneicosanoic acid (C21: 0)	42.905	1.770 ± 0.618^a,b^	0.693 ± 0.176^a^	0.584 ± 0.283^b^
cis‐11,14‐Eicosadienic acid (C20: 2)	43.687	0.467 ± 0.599^a,b^	0.213 ± 0.282^a^	0.218 ± 0.398^b^
Behenic acid (C22: 0)	43.765	0.982 ± 0.630^a,b^	0.539 ± 0.315^a^	0.443 ± 0.245^b^
cis‐8,11,14‐Eicosatrienoic acid (C20: 3n6)	44.345	0.355 ± 0.226^a,b^	0.175 ± 0.127^a^	0.134 ± 0.082^b^
Erucic acid (C22: 1n9)	44.499	1.781 ± 0.882^a,b^	0.821 ± 0.279^a,c^	0.762 ± 0.425^b,c^
cis‐11,14,17‐Eicosatrienoic acid (C20: 3n3)	45.101	0.598 ± 1.001^a,b^	0.158 ± 0.090^a^	0.172 ± 0.217^b^
Tricosanoic acid (C23: 0)	45.472	0.147 ± 0.128	0.148 ± 0.052	0.225 ± 0.396
Arachidonic acid (C20: 4n6)	45.752	0.616 ± 1.283^a,b^	0.160 ± 0.07^a,c^	0.097 ± 0.061^b,c^
cis‐13,16‐Docosadienoic acid (C22: 2)	46.113	0.175 ± 0.411	0.090 ± 0.069	0.159 ± 0.280
Lignoceric acid (C24: 0)	46.572	0.536 ± 0.436^a,b^	0.150 ± 0.106^a^	0.198 ± 0.242^b^
cis‐5,8,11,14,17‐Eicosapentaenoic acid (C20: 5n3)	47.445	0.494 ± 0.318^a,b^	0.132 ± 0.100^a^	0.169 ± 0.196^b^
Nervonic acid (C24: 1)	48.300	1.131 ± 1.477^a,b^	0.188 ± 0.119^a,c^	0.115 ± 0.098^b,c^
cis‐4,7,10,13,16,19‐Docosahexaenoic acid (C22: 6n3)	50.284	0.394 ± 0.247^a^	0.443 ± 0.430^b^	0.237 ± 0.325^a,b^
Petroselaidic acid (C18: 1*t*6)	38.836	—	—	—

Note

Equal letters indicate significant difference (*p* < .05).

Abbreviation: RT, retention time.

The most abundant fatty acid was oleic acid (C18: 1n9*c*), which showed no differences across the different milk production phases; followed by palmitic acid (C16: 0). The third most prevalent fatty acid was linoleic acid, which was significantly higher in transitional milk and mature milk. These values coincide with what has been reported in other studies analyzing the composition of fatty acids in breast milk of women from Canada (Ratnayake et al., 2014), Malaysia (Daud et al., 2013), Turkey (Samur et al., 2009), Brazil (De Souza Santos da Costa et al., 2016), China (Wan et al., 2010), the United States (Perrin et al., 2018), Chile (Duran & Masson, 2010), and Spain (Moltó‐Puigmartí et al., 2011), in which it was also found that the fatty acids with the highest concentration in human milk are oleic acid, palmitic acid, and linoleic acid.

Regarding saturated fatty acids, the values for palmitic acid (C16:0), which contributes approximately 10% of the energy ingested by the infant, were significantly higher in colostrum (21.80%, compared to 20.25% in transitional milk and 20.11% in mature milk; *p* < .05). This is interesting considering the important role this substance plays in building nervous tissue and its intervention in the processes of palmitoylation, gliogenesis, synaptogenesis, and myelination (Innis, 2016). Our results are comparable with those reported in Spanish women (colostrum: 22.71%; transitional milk: 21.70%; mature milk: 21.26%) (Moltó‐Puigmartí et al., 2011) and Brazilian mothers (colostrum: 26.09%; mature milk: 21.60%) (De Souza Santos da Costa et al., 2016), in which higher levels of palmitic acid were also observed in colostrum.

Among the monounsaturated fatty acids, the content of nervonic (C24:1) and erucic (C22:1 n9) acids was higher in colostrum, and significantly lower in transitional milk and mature milk (nervonic acid: 1.13% in colostrum; 0.18% in transitional milk; and 0.11% in mature milk; erucic acid: 1.78% in colostrum; 0.82% in transitional milk; and 0.76% in mature milk; *p* < .05). These results coincide with Moltó‐Puigmartí’s findings on women in Spain (nervonic acid: 0.32% in colostrum; 0.08% in transitional milk; and 0.05% in mature milk; erucic acid: 0.27% in colostrum; 0.12% in transitional milk; and 0.10% in mature milk). These fatty acids are important for the metabolic role they play in myelin biosynthesis and in the development of the newborn's nervous system (Li et al., 2019).

#### 3.4.1 Essential fatty acid content in breast milk

Linoleic acid was the most abundant EFA in all three stages of human milk. The *n*−6 series polyunsaturated acids—linoleic acid and γ‐linolenic acid (C18:3 n6)—showed a similar concentration in transitional milk and mature milk, which was significantly higher than in the colostrum stage (*p* < .05). Arachidonic acid (AA), on the other hand, was significantly higher in colostrum, decreasing in mature milk, as were linolenic acid (*p* < .05); eicosapentaenoic acid (EPA), and total *n*−3 series polyunsaturated fatty acids, all observed to be in higher concentration in colostrum (*p* < .05). Docosahexaenoic acid (DHA) content was significantly higher in both transitional milk and colostrum, than in mature milk (*p* < .05) (Tables 3 and 4). EFAs and their LC‐PUFAs are vital to the newborn due to their role in visual, brain and cognitive development, where they participate in numerous neuronal processes, from effects on the fluidity of the membrane as some of its component phospholipids, to their involvement in regulating gene expression (Campoy et al., 2012; Vega et al., 2012).

**TABLE 4:** Content of EFAs, LC‐PUFAs, and industrially produced TFAs in colostrum, transitional milk, and mature milk (*n* = 33) (%)

	Colostrum	Transitional milk	Mature milk
EFAs and LC‐PUFAs			
Linoleic acid (*n*−6) (%)	13.179 ± 3.247^a,b^	15.540 ± 3.609^a^	15.660 ± 4.085^b^
γ‐Linolenic acid (*n*−6) (%)	1.087 ± 0.580^a,b^	1.402 ± 0.573^a^	1.554 ± 0.651^b^
Arachidonic acid (*n*−6) (%)	0.616 ± 1.283ª^,b^	0.160 ± 0.07ª^,c^	0.097 ± 0.061^b,c^
Linolenic acid (*n*−3) (%)	0.699 ± 1.085^a,b^	0.170 ± 0.095^a^	0.665 ± 1.749^b^
Eicosapentaenoic acid (*n*−3) (%)	0.494 ± 0.318^a,b^	0.132 ± 0.100^a^	0.169 ± 0.196^b^
Docosahexaenoic acid (*n*−3) (%)	0.394 ± 0.247^a^	0.443 ± 0.430^b^	0.237 ± 0.325^a,b^
Total *n*−3 fatty acids (%)	1.586 ± 1.195^a,b^	0.744 ± 0.502^a^	1.071 ± 1.853^b^
Total *n*−6 fatty acids (%)	14.266 ± 3.203^a,b^	16.942 ± 4.117^a^	17.213 ± 4.220^b^
Industrially produced TFAs			
Elaidic acid (%)	1.334 ± 1.699^a,b^	0.503 ± 0.846^a^	0.585 ± 1.042^b^
Linoelaidic acid (%)	0.196 ± 0.182	0.246 ± 0.232	0.361 ± 0.535
Total TFA (%)	1.529 ± 1.648^a^	0.748 ± 1.033^a^	0.945 ± 1.368

Note

Equal letters indicate significant difference (*p* < .05).

Abbreviations: EFAs, essential fatty acids; LC‐PUFAs, long‐chain polyunsaturated fatty acids; TFAs, trans fatty acids.

Comparing the EFA content in colostrum from this study with others, we note that the milk of these Mexican mothers had higher EPA values than the mothers of Brazil (0.49% vs. 0.08%). However, they presented lower levels of linoleic acid and DHA than Brazilian women (linoleic acid: 13.17% vs. 18.05%; DHA: 0.39% vs. 0.92%) (De Souza Santos da Costa et al., 2016).

The AA content was lower than what was found in Spanish women in the three stages of milk production (colostrum: 0.61% vs. 0.92%; transitional milk: 0.16% vs. 0.62%; and mature milk: 0.097% vs. 0.49%). But they showed a similar pattern, with content significantly higher in colostrum and decreasing in the subsequent stages (Moltó‐Puigmartí et al., 2011).

In mature milk, differences in EFA concentration were also found compared to other studies, showing variability in the composition of milk of women from different countries (Daud et al., 2013; De Souza Santos da Costa et al., 2016; Deng et al., 2018; Duran & Masson, 2010; Gómez‐Cortés & De La Fuente, 2017; Krešić et al., 2013; Moltó‐Puigmartí et al., 2011; Perrin et al., 2018; Ratnayake et al., 2014; Samur et al., 2009). This was also the case with DHA (0.23%), which showed higher concentrations in this study than in a study of Turkish lactating women (0.15%) (Samur et al., 2009), Chinese (0.19%) (Deng et al., 2018), Americans (0.04%) (Perrin et al., 2018), Chileans (0.20%) (Duran & Masson, 2010), and Brazilians (0.17%) (De Souza Santos da Costa et al., 2016). Likewise, DHA (0.23%) was lower for Mexican mothers than for mothers in Canada (0.3%) (Ratnayake et al., 2014), Malaysia (0.82%) (Daud et al., 2013), Nigeria (0.27%) (Gómez‐Cortés & De La Fuente, 2017), and Spain (0.35%) (Moltó‐Puigmartí et al., 2011). This may be due to differences in diet since these fatty acids and their precursors come from the diet.

#### 3.4.2 Trans fatty acid content in breast milk

The study looked for three TFAs–elaidic, linoelaidic, and petroselaidic fatty acids—but found only elaidic and linoelaidic acids in studied samples.

Elaidic acid was the most abundant in all three stages, representing 87.2% of the total TFA in colostrum, 67.2% in transitional milk, and 61.9% in mature milk. This fatty acid was found in significantly higher amounts in colostrum (1.334%; *p* < .05), than in either transitional milk (0.503%) or mature milk (0.585%). Linoelaidic acid accounted for 12.8% of total TFAs in colostrum, 32.8% in transitional milk, and 38.1% in mature milk, but with no differences in its content according to the stage of milk production (*p* > .05). Total TFA content was higher in colostrum and was significantly different from the values found in transitional milk (*p* < .05) (Table 4). This could have important implications in this first stage of development, due to the probable damage that may be caused by the presence of TFAs, including changes in the fluidity of the phospholipid membrane and with interference in EFA metabolism.

Petroselaidic acid was not identified in any sample, because its concentration in food is very small and it is below the detection limit of the method used. This industrially produced TFA was identified in other studies in which it was found in small quantities in breast milk and coeluted with other isomers such as 18:1 *t*8 (18:1 *t*6+8) (De Souza Santos da Costa et al., 2016) and with 18:1 *t*7 and 18:1 *t*8 (18:1 *t*6+7 + 8) (Gómez‐Cortés & De La Fuente, 2017).

There are few studies that analyze industrially produced TFAs in human milk, and even fewer that study the presence of these fatty acids in all three stages of lactation. However, the findings of the present study coincide with some previously reported works, in which the industrially produced TFA was found in the highest concentrations of iselaidic acid, followed by linoelaidic acid (De Souza Santos da Costa et al., 2016; Duran & Masson, 2010; Gómez‐Cortés & De La Fuente, 2017; Krešić et al., 2013; Ratnayake et al., 2014; Samur et al., 2009). In contrast, in a study conducted in Malaysian women, the TFA in the highest concentration was linoelaidic acid (1.44%), followed by petroselaidic (0.66%), palmitoelaidic (0.46%), vaccenic acid (this being of natural origin) fatty acids (0.15%) and, lastly, elaidic acid (0.22%) (Daud et al., 2013). It should be noted that the common literature indicates that the most prevalent TFA in breast milk is elaidic acid, which is also the most prevalent industrially produced TFA in food (Hauff & Vetter, 2009; Krešić et al., 2013; De Souza et al., 2015; Villalpando, 2007).

The amounts found are also comparable with the values of elaidic acid found in colostrum (1.91%) and mature milk (0.55%) of Brazilian adolescent women (De Souza Santos da Costa et al., 2016); with concentrations of elaidic acid (1.96%) and linoelaidic acid (0.15%) in mature milk of women in Turkey (Samur et al., 2009); of elaidic acid (1.95%) and linoelaidic acid (0.33%) in mature milk of Chilean mothers (Duran & Masson, 2010) and of elaidic acid (1.3%) and linoelaidic acid (0.4%) in mature milk of Canadian women (Ratnayake et al., 2014).

The total TFA found in the mature milk of the samples analyzed in this study was lower (0.95%) than that reported in the mature milk of women in Canada (1.9%) (Ratnayake et al., 2014), Turkey (2.11%) (Samur et al., 2009), Malaysia (1.66%) (Daud et al., 2013), Croatia (2.3%) (Krešić et al., 2013), Chile (3.68%) (Duran & Masson, 2010), and Brazil (2.23%) (De Souza Santos da Costa et al., 2016). It was higher than that of lactating women in China (0.77%) (Deng et al., 2018). While similar results were observed in women in the United States (1.09%) (Perrin et al., 2018), this could be due to the geographic proximity and therefore similarity with respect to diet.

Considering that most of the existing studies are carried out on mature milk, it is difficult to draw comparisons regarding colostrum and transitional milk. However, taking advantage of some studies in which colostrum is analyzed, we can say that the content of TFAs was lower in this study in Mexico than in the colostrum of Brazilian women (1.53% vs. 2.46%) (De Souza Santos da Costa et al., 2016), but higher than that of Spanish mothers (1.53% vs. 0.45%) (Moltó‐Puigmartí et al., 2011). Likewise, the concentration of TFAs in the transitional milk of the samples analyzed was also higher (0.74%) than those observed in women in Spain (0.51%) (Moltó‐Puigmartí et al., 2011).

For comparative purposes, in addition to determining the content of TFAs C18:1t in breast milk using the methodology described above, the Craig‐Schmidt formula was applied using the values of the estimated maternal intake of TFAs. In 1984, Craig‐Schmidt published a work suggesting an equation for estimating the quantity of TFAs in breast milk based on the estimated dietary intake (Craig‐Schmidt et al., 1984). The use of this formula has been extended to some current studies. We found that although the elaidic acid content of colostrum estimated using the Craig‐Schmidt formula was higher than our own finding, it did not differ significantly (1.75% vs. 1.33%). In transitional milk and mature milk, the formula resulted in significantly higher values (transitional milk: 1.76%; mature milk: 1.78%), than what were determined in the present study (transitional milk: 0.50%; mature milk: 0.58%).

### 3.5 Fatty acid content in breast milk, according to anthropometric measurements of the mother

The content of TFAs and EFAs in breast milk (colostrum, transitional milk, and mature milk) was compared against anthropometric measurements, according to WHO criteria (Organización Mundial de la Salud, 2000). The mothers were classified into two groups according to their BMI: Group 1: low and normal weight (BMI ≤24.9) and Group 2: overweight and obese (BMI ≥25.0). Mothers were also stratified according to their percentage of body fat: Group 1, decreased and healthy (≤32.99%) and Group 2, high and very high (≥33.0) (Gallagher et al., 2000). The anthropometric measurements of the mother (BMI and percentage of body fat) were not found to correlate with the composition of TFAs or EFAs in breast milk in any of the three stages. This is comparable to another study in which there was also no relationship between the content of 18:1 TFAs in milk and the weight and BMI of Brazilian women (De Souza Santos da Costa et al., 2016).

### 3.6 Relationship between maternal intake and the content of trans fatty acids and essential fatty acids in milk

The results do not show a correlation between the content of TFAs in milk and the intake of TFAs, either expressed in grams or expressed as a percentage of the total caloric value of the diet obtained from the analysis of the 24HR recall (*p* > .05). Nor was the content of TFAs in milk found to correlate with the percentage of lipids, proteins, and carbohydrates consumed in the diet or with energy consumption.

These results coincide with those reported by Samur et al. (2009), who studied the composition of TFAs and its association with the diet of lactating women in Turkey also obtained using the 24HR. Although they found that there was no significant correlation between TFAs in milk and diet, they reported that mothers who presented a high level of trans isomers in their milk consumed significantly higher amounts of products rich in TFAs. There are other studies which have suggested a relationship between the TFA content in breast milk and TFA consumption by the mother: although Gomez Cortés and De la Fuente (Gómez‐Cortés & De La Fuente, 2017) did not directly study mothers’ diet, they studied and compared the composition of milk produced by rural and urban women, finding that the percentage of elaidic acid was significantly higher in women in urban areas (0.07% vs. 0.14%, respectively). Their assumption was that these women consume more TFAs because they had more access to industrialized food. A recent study by Perrin et al. (2018) analyzed differences in the fatty acid composition of the milk of mothers with different eating habits (vegan, vegetarian, and omnivorous) in lactating women in the United States, finding a total TFA concentration of 0.44% in the milk of vegan women, significantly lower than what was determined among vegetarian (0.66%) and omnivorous (1.09%) women. A study by Ratnayake et al., 2014 (Ratnayake et al., 2014) of Canadian women's breast milk samples collected in 2009–2011 estimated TFA consumption using the Craig‐Schmidt formula (1984) (Craig‐Schmidt et al., 1984), and the values of TFAs in breast milk (no food survey was applied in this study). They reported a progressive decrease in estimated TFA consumption from 1998 to 2011, which they assumed was the result of government policies to regulate TFA content in food, including passage of the mandatory labeling law in 2005 and the establishment of the maximum levels used during 2007.

The relation between intake of TFAs, energy and macronutrient consumption, and EFA content in breast milk was also analyzed. It was found that the higher the calorie intake, the greater the likelihood of a higher content of linolenic acid in colostrum (OR = 1.004; *p* = .039). Protein consumption was associated with a lower content of linoleic acid in colostrum (odds ratio (OR) = 0.963; *p* = .031). The average intake of carbohydrates during all three stages of breastfeeding was associated with a lower content of total *n*−3 fatty acids (OR = 0.998; *p* = .047), and the average intake of protein was associated with a higher content of TFA (OR = 2.207; *p* = .044).

These results indicate that there are aspects of a mother's diet that may correlate with the content of fatty acids in breast milk, and that this correlation may be significant. This last aspect merits further study.

### 3.7 Relationship between the content of essential fatty acids and trans fatty acids in colostrum, transitional milk, and mature milk

The correlation between EFAs and TFAs was evaluated in breast milk (Table 5). In colostrum, among the *n*−6 series fatty acids, linoleic acid showed an inverse correlation with elaidic acid content and with total TFA content (*p* < .05); while AA, γ‐linolenic acid, and LCPUFA *n*−6 (γ‐linolenic acid + arachidonic acid) showed a positive correlation with both elaidic acid and total TFAs. Among the *n*−3 series fatty acids, linolenic acid and total *n*−3 series fatty acids correlated positively with elaidic acid content and total TFA content (*p* < .05). However, there was an inverse correlation between DHA content and both elaidic acid content and total TFAs. In the case of transitional milk, linoleic acid, AA, and total *n*−6 were negatively associated with linoelaidic acid (*p* < .05). However, with the exception of the EPA fatty acid, the *n*−3 family: linolenic acid, DHA, the sum of LC‐PUFA *n*−3 (EPA + DHA) and total *n*−3, showed a positive correlation with the levels of elaidic acid, linoelaidic acid, and total of TFAs (*p* < .05). Finally, in mature milk, among the *n*−6 series fatty acids, linoleic acid showed a negative correlation with total TFAs and AA also a negative correlation with the content of elaidic and linoelaidic acid; while γ‐linolenic acid and LC‐PUFA *n*−6 had a positive correlation: γ‐linolenic acid with linoelaidic acid and the total of TFAs, and LC‐PUFA *n*−6 with linoelaidic acid content (*p* < .05). Furthermore, among the *n*−3 series fatty acids, linolenic acid, EPA, LC‐PUFA *n*−3, and total *n*−3 showed a positive correlation with the levels of linoelaidic acid and total TFAs; the total of *n*−3 also showed a positive correlation with elaidic acid content (*p* < .05) (Table 5).

**TABLE 5:** Relationship between the content of EFAs and TFAs in colostrum, transitional milk, and mature milk

	Elaidic acid		Linoelaidic acid		Total TFA	
	*R*	*p*	*R*	*p*	*R*	*p*
Colostrum						
Linoleic acid	−.670	.000*	.268	.131	−.661	.000*
Arachidonic acid	.764	.000*	−.259	.146	.758	.000*
γ‐Linolenic acid	.612	.000*	−.086	.634	.621	.000*
Linolenic acid	.844	.000*	−.294	.097	.837	.000*
EPA	.222	.213	−.230	.198	.204	.255
DHA	−.422	.014*	.482	.005*	−.382	.028*
Total *n*−6	−.280	.114	.163	.364	−.271	.127
Total *n*−3	.738	.000*	−.228	.202	.735	.000*
LC‐PUFA *n*−6^a^	.735	.000*	−.210	.240	.734	.000*
LC‐PUFA *n*−3^b^	−.086	.634	.118	.513	−.076	.675
Transitional milk						
Linoleic acid	−.295	.096	−.385	.027*	−.328	.063
Arachidonic acid	−.017	.924	−.410	.018*	−.106	.556
γ‐Linolenic acid	−.170	.345	−.254	.154	−.196	.274
Linolenic acid	.844	.000*	.782	.000*	.867	.000*
EPA	−0.245	.170	−.159	.377	−.236	.186
DHA	.615	.000*	.843	.000*	.693	.000*
Total *n*−6	−.281	.113	−.379	.030*	−.315	.074
Total *n*−3	.638	.000*	.837	.000*	.710	.000*
LC‐PUFA *n*−6^a^	−.166	.356	−.300	.090	−.203	.256
LC‐PUFA *n*−3^b^	.537	.001*	.774	.000*	.613	.000*
Mature milk						
Linoleic acid	−0.329	.62	−.323	.066	−.377	.031*
Arachidonic acid	−0.364	.037*	−.624	.000*	−.521	.002
γ‐Linolenic acid	.253	.155	.476	.005*	.379	.030*
Linolenic acid	.334	.057	.840	.000*	.583	.000*
EPA	.305	.084	.602	.000*	.468	.006*
DHA	.134	.456	.151	.401	.161	.370
Total *n*−6	−.284	.110	−.248	.165	−.313	.076
Total *n*−3	.371	.033*	.883	.000*	.628	.000*
LC‐PUFA *n*−6^a^	.225	.207	.430	.013*	.340	.053
LC‐PUFA *n*−3^b^	.325	.065	.525	.002*	.453	.008*

Abbreviations: DHA, docosahexaenoic acid; EFAs, essential fatty acids; EPA, eicosapentaenoic acid; LC‐PUFAs, long‐chain polyunsaturated fatty acids; TFAs, trans fatty acids.

^a^ LC‐PUFA *n*−6: γ‐linolenic acid + Arachidonic acid.

^b^ LC‐PUFA *n*−3: EPA + DHA.

* *p* < .05.

Similar correlations were found between linolenic acid and total fatty acids of the *n*−3 series, because linolenic acid is the most prevalent fatty acid of the *n*−3 family. There were also similarities between the behavior of elaidic acid and the sum of total TFAs, since elaidic acid makes up 87.2% of total TFAs.

The few studies that exist on the correlation between TFAs and EFAs in mature milk do not coincide. Krešić et al. (2013) observed an inverse relationship between linoleic acid, linolenic fatty acids, EPA, DHA, and total LC‐PUFA, with total TFAs in mature milk. De Souza Santos da Costa et al. (2016), on the other hand, looked for a correlation between the most abundant TFAs, elaidic acid, and concentrations of LC‐PUFAs in mature milk, but were unable to find one. Samur et al., 2009 (Samur et al., 2009) failed to observe a correlation between total elaidic acid or total TFAs and linoleic, linolenic, total *n*−3, and total *n*−6 fatty acids.

In our study, we did find a correlation between TFAs and EFA content in all three stages of maternal milk, although this correlation was, on many occasions, positive. In colostrum, despite the fact that linolenic acid was positively associated with elaidic acid content, the LC‐PUFA of greater importance to the newborn's visual and nervous system development, DHA, was inversely related. This could be due to the negative effect of the presence of TFAs on the metabolism of EFAs, since TFAs compete for the same enzymes (∆5 and ∆6 desaturases), blocking them and preventing DHA biosynthesis from taking place in the mother's body, which can change the composition of breast milk (Krešić et al., 2013; Kummerow, 2009; Villalpando, 2007).

De Souza Santos da Costa et al. (2016) analyzed the colostrum of 54 Brazilian adolescents and found that the content of LC‐PUFA *n*−3 was negatively associated with the total concentration of TFAs. In our study, an inverse relationship was found with the DHA content in colostrum, which decreased significantly as total TFAs increased.

Our findings show that although the increase in TFAs in both the maternal diet and milk may be accompanied by the increase in some EFAs in milk, it may also be inversely related to the presence of EFAs such as DHA in important stages of development, which could have an effect on the metabolism and development of the newborn.

The effect of TFAs present in milk on the composition of the other fatty acids and the effect that TFAs in the milk may have on the newborn's body, although they correlate positively with some EFAs, warrant further study.

In perspective, more studies are needed on the composition of TFAs in breast milk, and fresh research on its consumption in newborns. It would be useful to analyze the impact that the content of TFAs in human milk has on health, and the impact that it has on the composition of fatty acids. Although in the interest of analyzing TFAs in breast milk, both industrially and naturally produced TFAs are evaluated, it is important to make a clear distinction, because they do not have the same involvement in the body. It has been recognized that the inclusion of a moderate amount of TFAs of natural origin has a positive effect on health, while industrially produced TFAs are attributed with negative effects (Daud et al., 2013; De Souza Santos da Costa et al., 2016; Deng et al., 2018; Duran & Masson, 2010; Gómez‐Cortés & De La Fuente, 2017).

The tables showing the TFA composition of food consumed frequently in various regions—including Mexico—must be kept continually up to date. For the moment, these tables afford a limited approach and may contribute to the underestimation of the consumption of TFAs as well as the association between TFAs in breast milk and TFAs in the mothers’ diets. Likewise, a validated tool is required for collecting information on the intake of trans fats, which would allow for a more accurate estimate of their consumption.

## 4 CONCLUSION

In this study, TFAs were found in maternal milk. The trans isomer in the highest concentration in all three stages of milk was elaidic acid (C18:1 *t*9*)*. In the analyzed samples, TFA concentrations were significantly higher in colostrum than in transitional or mature milk, and elaidic acid and total TFA levels showed an inverse correlation with DHA concentrations in milk from the same stage. The concentration of TFAs has a relationship with the composition of the EFAs in milk, which could have a negative effect on the development of the newborn.

## CONFLICT OF INTEREST

The authors declare that they do not have any conflict of interest.

## ETHICAL CONSIDERATIONS

This work was carried out in keeping with the ethical principles for medical research involving human subjects adopted in the 18th World Medical Assembly, Helsinki, Finland, June 1964. The study was approved by the Bioethics Committee of the School of Natural Sciences, UAQ. The study does not involve any risk, since the expression of breast milk is a common procedure. Participants included in the study received an evaluation of their body composition and a personalized dietary plan at the end of their participation in the study, as well as advice on breastfeeding beginning with the first meeting.

## INFORMED CONSENT

The women were invited to participate and received an explanation of the study. Those who met the inclusion criteria and accepted were given an informed consent form to sign.
